# An immune-related lncRNA signature for the prognosis of pancreatic adenocarcinoma

**DOI:** 10.18632/aging.203323

**Published:** 2021-07-20

**Authors:** Bing Qi, Han Liu, Qi Zhou, Li Ji, Xueying Shi, Yushan Wei, Yajun Gu, Akio Mizushima, Shilin Xia

**Affiliations:** 1 Department of General Surgery, The First Affiliated Hospital of Dalian Medical University, Dalian 116011, Liaoning, China; 2 College of Stomatology, Dalian Medical University, Dalian 116044, Liaoning, China; 3 Institute (College) of Integrative Medicine, Dalian Medical University, Dalian 116044, Liaoning, China; 4 Department of Gastroenterology, Liaoning University of Traditional Chinese Medicine, Shenyang 110032, Liaoning, China; 5 Department of Scientific Research, The First Affiliated Hospital of Dalian Medical University, Dalian 116011, Liaoning, China; 6 School of Medical Laboratory, Tianjin Medical University, Tianjin 300000, Tianjin, China; 7 Department of Palliative Medicine, Graduate School of Medicine, Juntendo University, Tokyo 1138421, Japan; 8 Clinical Laboratory of Integrative Medicine, The First Affiliated Hospital of Dalian Medical University, Dalian 116011, Liaoning, China

**Keywords:** pancreatic adenocarcinoma, lncRNA, signature, prognosis, immune infiltration

## Abstract

Recent evidence suggests that aberrant expression of long non-coding RNA (lncRNA) can drive the initiation and progression of malignancies. However, little is known about the prognostic potential of lncRNA. We aimed at constructing a lncRNA-based signature to improve the prognosis prediction of pancreatic adenocarcinoma (PAAD). The PAAD samples with clinical information were obtained from The Cancer Genome Atlas and International Cancer Genome Consortium. We established an eight-IRlncRNA signature in a training cohort. The prognostic value of eight-IRlncRNA signature was validated in two distinct cohorts when compared to other four prognostic models. We continued to analyze its independence in subgroups by univariate and multivariate Cox regression. We constructed a nomogram for clinicopathologic features and 1-, 3-, and 5-year overall survival performance. Moreover, Gene set enrichment analysis and Gene Set Variation Analysis distinguished the typical functions between high- and low-risk groups. In addition, we further observed the different correlations of immune cell between eight IRlncRNAs. Eight-IRlncRNA signature appears to be a good performer to predict the survival capability of PAAD patients, and the nomogram will enable PAAD patients to be more accurately managed in clinical practice.

## INTRODUCTION

Pancreatic cancer is an aggressive malignant neoplasm with a poor prognosis [1, 2]. Pancreatic adenocarcinoma (PAAD) comprises 85% of all pancreatic cancer cases, and therefore PAAD is the focus of pancreatic cancer studies [3]. PAAD can be derived from the premalignant pancreatic lesions, which is referred to as pancreatic intraepithelial neoplasia [4]. More advanced lesions contribute to a stepwise process of adenocarcinomas with both local invasion and distant metastasis. The progression of PAAD is accompanied by an extensive stromal reaction, typically as desmoplasia, which results in hypoxic microenvironment and immune evasion [5]. Also, the initiation and progression of PAAD are associated with accumulating genetic alterations, such as CDKN2A, KRAS, and TP53 [6]. The precise molecular origins of human PAAD remain only partially understood.

Extensive research has shown that the majority of genomic products are transcribed in long non-coding RNAs (lncRNAs), which have been found to play a crucial role in the biological process of cancer [7, 8]. Non-coding sequence in genome was historically regarded as junk DNA. High-throughput technology allowed an in-depth data mining to identify lncRNA. lncRNA, the transcript of more than 200 nucleotides, has diverse functions via four modes including decoy, signal, scaffold, and guide [8]. Recent studies have explored some oncogenic lncRNAs associated with poor prognosis of PAAD, such as HOTAIR [9], PVT1 [10], and SNHG8 [11]. Despite great advances in understanding that lncRNAs may act as signals, decoys, scaffolds, or guides to interact with other molecules, emerging roles of lncRNA on the tumorigenesis and prognosis of PAAD await elucidation.

Here, we have identified immune-related lncRNA (IRlncRNA) harboring the ability to involve in the immune response of PAAD. An eight-IRlncRNA signature is constructed via an analysis of 435 PAAD patients in three distinct cohorts from The Cancer Genome Atlas (TCGA) and International Cancer Genome Consortium (ICGC). Then, we performed a nomogram to assess the independence of eight-IRlncRNA signature in subgroups relevant to clinicopathologic features. Moreover, we characterized the role of eight-IRlncRNA signature on immune response by multiple analyses, including immune infiltration analysis, overall survival analysis, and correlation analysis between eight IRlncRNAs and immune cells.

The serial analyses with robust statistical power have provided biological insights of IRlncRNAs and describe prognostic value of the signature in patients with PAAD. The identification of the signature may become beneficial to the clinical management of PAAD.

## RESULTS

### Identification of prognostic IRGs and IRlncRNAs in patients with PAAD

A total of 1,900 IRGs were collected from the ImmPort database. Among 1,900 IRGs, 332 prognostic IRGs were obtained to predict OS in PAAD patients from TCGA database. Subsequently, prognostic IRGs were analyzed with GO and KEGG enrichment analyses. We found that IRGs were enriched in leukocyte migration, peptide secretion, innate immune response, external side of plasma membrane, collagen-containing extracellular matrix, receptor ligand activity, and cytokine activity. The main enrichment pathways in KEGG were cytokine-cytokine receptor interaction, neuroactive ligand-receptor interaction, and JAK-STAT signaling pathway (Supplementary Figure 1).

The co-expression analysis was used to identify 1,471 IRlncRNAs, which were potentially involved in immune regulation. To explore prognostic value of IRlncRNA in patients with PAAD, we performed univariate Cox regression to identify prognostic IRlncRNA. We found that there were 132 prognostic IRlncRNAs among 1,471 IRlncRNAs (Supplementary Table 1). Furthermore, we performed a functional enrichment analysis for 132 lncRNA-targeted genes. We found significant enrichment in the following categories: T cell activation, extracellular structure organization, extracellular structure organization, cell-substrate junction, cell adhesion molecule binding (Supplementary Figure 2). The above results suggested that IRlncRNAs were implicated in the occurrence and development of PAAD by improving an interaction between tumor cell and immunocyte.

### An IRlncRNA-based signature for prognosis prediction in PAAD patients

**Figure 1:**
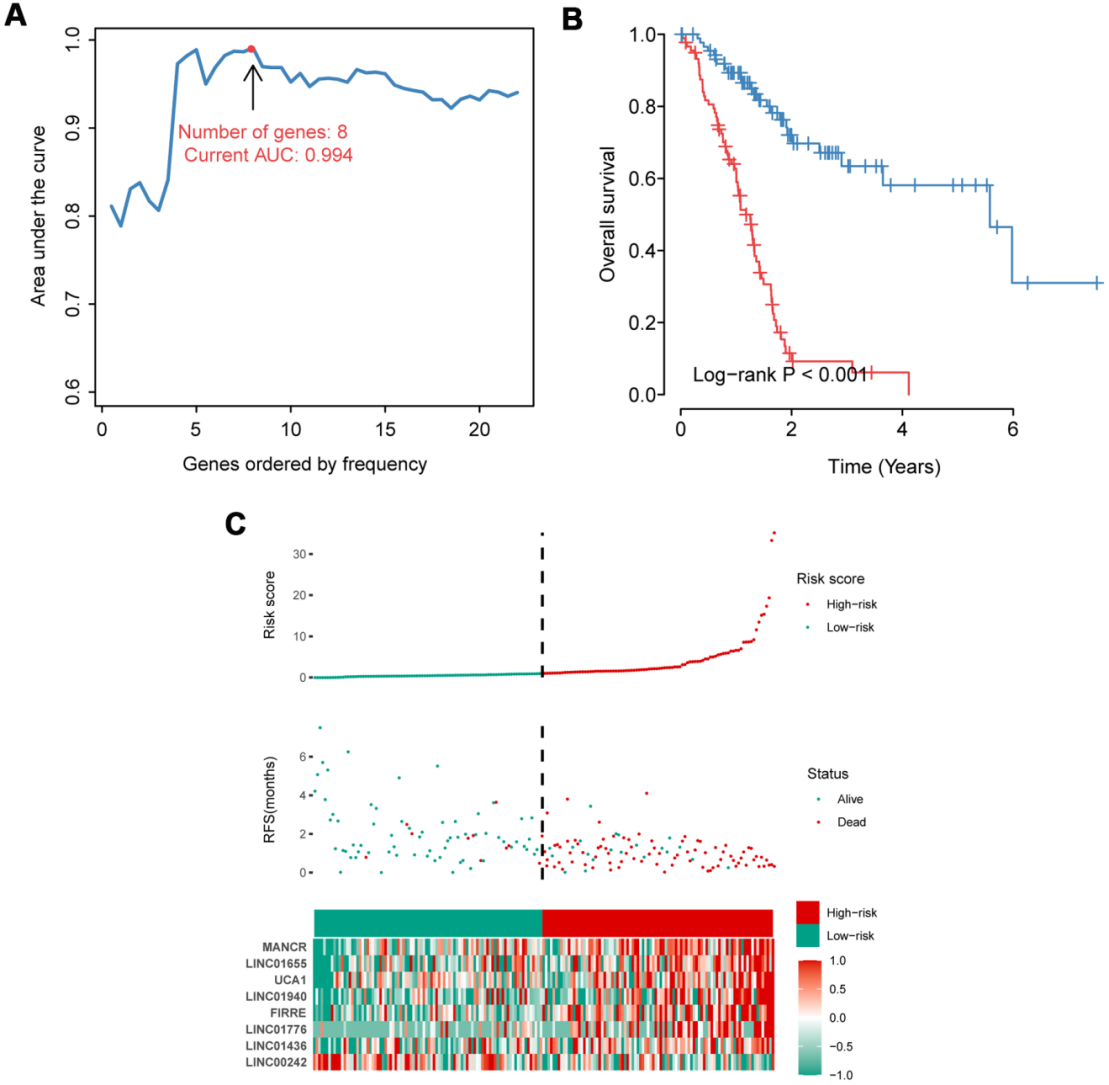
**Construction and evaluation of the eight-IRlncRNA signature.** (**A**) Eight-IRlncRNA signature constructed by iterative Lasso Cox regression. (**B**) Kaplan-Meier curves for the high- and low-risk groups. (**C**) Risk score analysis including distribution, survival status, and the heatmap.

Among 132 IRlncRNAs with prognostic value, we integrated eight IRlncRNAs to derive a lncRNA-based signature for a prognosis prediction using an iterative Lasso Cox regression analysis (Figure 1). ROC analysis displayed an advantage of this eight-IRlncRNA signature with AUC=0.994 (Figure 1A). The PAAD patients were divided into high- and low-risk groups by the median=1.041. Kaplan-Meier survival analysis showed that the OS of PAAD patients in high-risk group was significantly lower than that in low-risk group (*P*< 0.001, Figure 1B). The number of deaths in high-risk group was obviously higher than that in low-risk group (Figure 1C). In total, the eight-IRlncRNA signature, which divided PAAD patients into high- and low-risk groups, was of great significance in the prediction of PAAD prognosis.

### Validation of the eight-IRlncRNA signature

**Figure 2:**
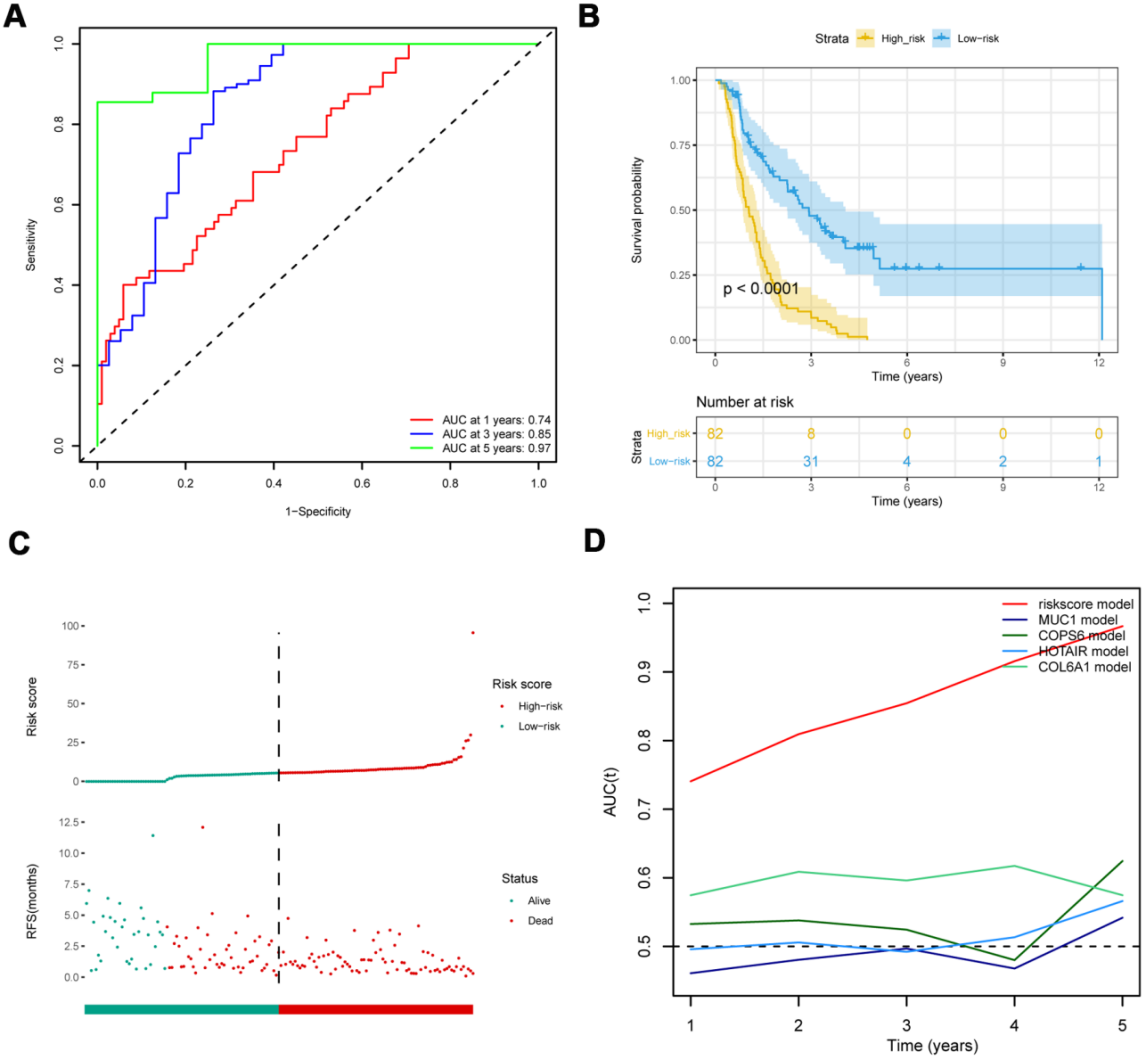
**Comparison with other prognostic biomarkers and validation of eight-IRlncRNA signature in the validation cohort (PACA-CA).** (**A**) ROC curve of eight-IRlncRNA signature for 1, 3, 5- year survival. (**B**) Kaplan-Meier curves for the high- and low-risk groups. (**C**) Risk score distribution and the survival status for patients. (**D**) Time-dependent ROC curve for the eight-IRlncRNA signature compared with other prognosis biomarkers.

To further assess the predictive potential of eight-IRlncRNA signature, we examined the prediction value of eight-IRlncRNA signature using two independent cohorts of 164 samples from PACA-CA data set and 90 samples from PACA-AU data set. In PACA-CA, ROC analysis showed that the prognostic accuracy of eight-IRlncRNA signature was 0.74 at 1 year, 0.85 at 3 years and 0.97 at 5 years (Figure 2A), indicating that eight-IRlncRNA signature had a good prediction efficiency in PACA-CA data set. Kaplan-Meier survival analysis showed that the OS of patients in high-risk group was significantly lower than that in low-risk group (*P* < 0.001, Figure 2B). The distribution of risk scores and survival status of eight-IRlncRNA signature revealed that number of deaths in high-risk group was significantly larger than that in low-risk group (Figure 2C).

The ROC analysis showed that the AUC of eight-IRlncRNA signature was higher than that of other biomarkers (Figure 2D), indicating that the eight-IRlncRNA signature was a better prognostic biomarker with higher robustness and reliability. The results of the other cohort from PACA-AU data set were basically consistent with that of PACA-CA data set (Supplementary Figure 3). These findings demonstrated the eight-IRlncRNA signature was capable of predicting the OS of PAAD patients in different cohorts.

### Subgroup analysis of the prognostic value of eight-IRlncRNA signature

**Figure 3:**
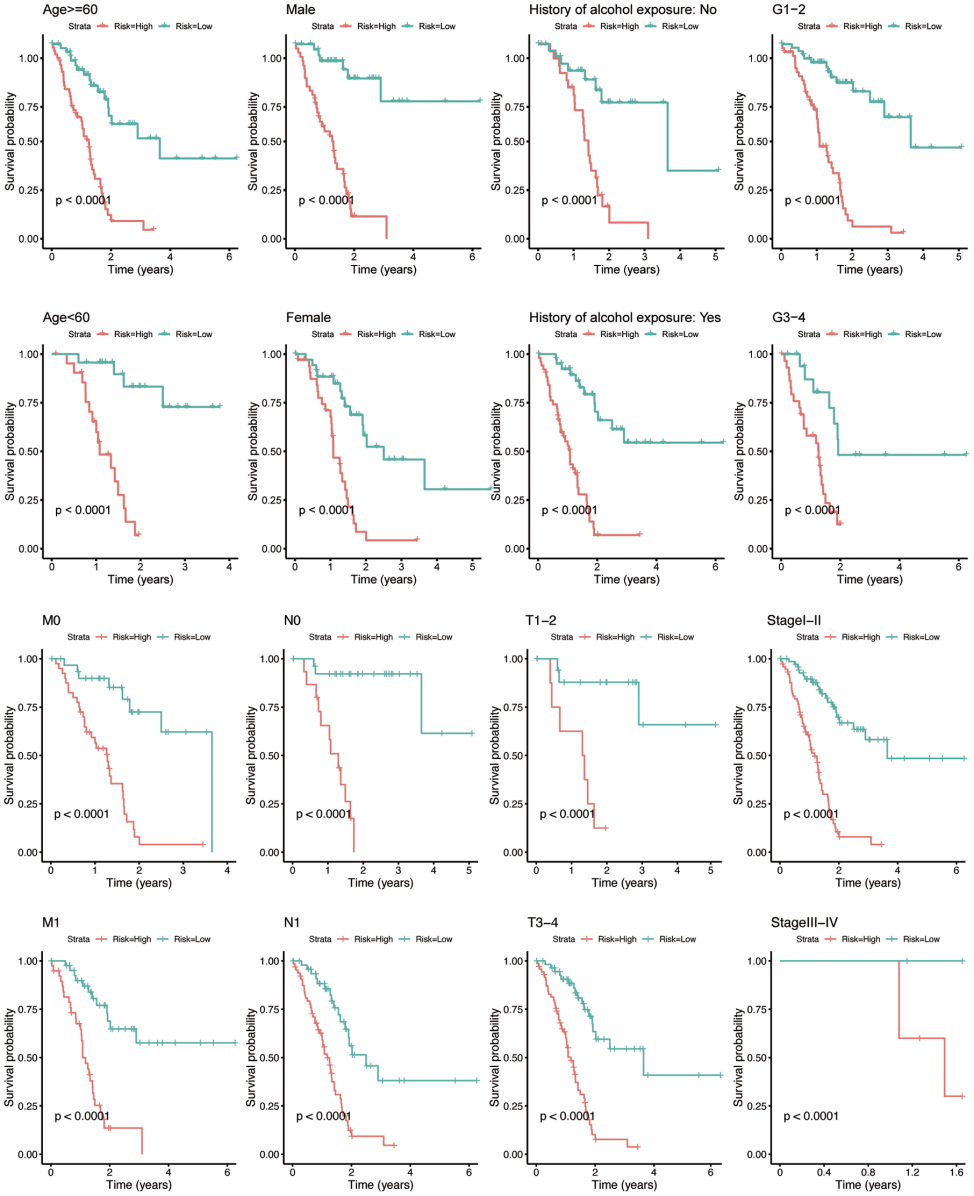
**Kaplan-Meier analysis for PAAD patients between subgroups according to clinicopathological features, including age, gender, history of alcohol exposure, grade, M-, N-, T- classification, and AJCC stage.** The X-axis indicates the time in years. The red curve represents the high-risk group, and the blue curve represents the low-risk group.

To investigate whether the prognostic value of eight-IRlncRNA signature was independent from conventional clinicopathologic characteristics, we categorized PAAD patients into distinct subgroups according to eight clinicopathologic characteristics, including age, gender, history of alcohol exposure, pathological grade, TNM stages, and AJCC stage (Figure 3). In all eight groups, the low-risk group signified a longer OS of the PAAD patients (*P* < 0.0001). The results revealed an independence of prognostic value of eight-IRlncRNA signature from clinicopathologic features, indicating that the eight-IRlncRNA signature was an independent indicator to predict the prognosis of PAAD patients.

### Independence of the prognostic value of eight-IRlncRNA signature

**Figure 4:**
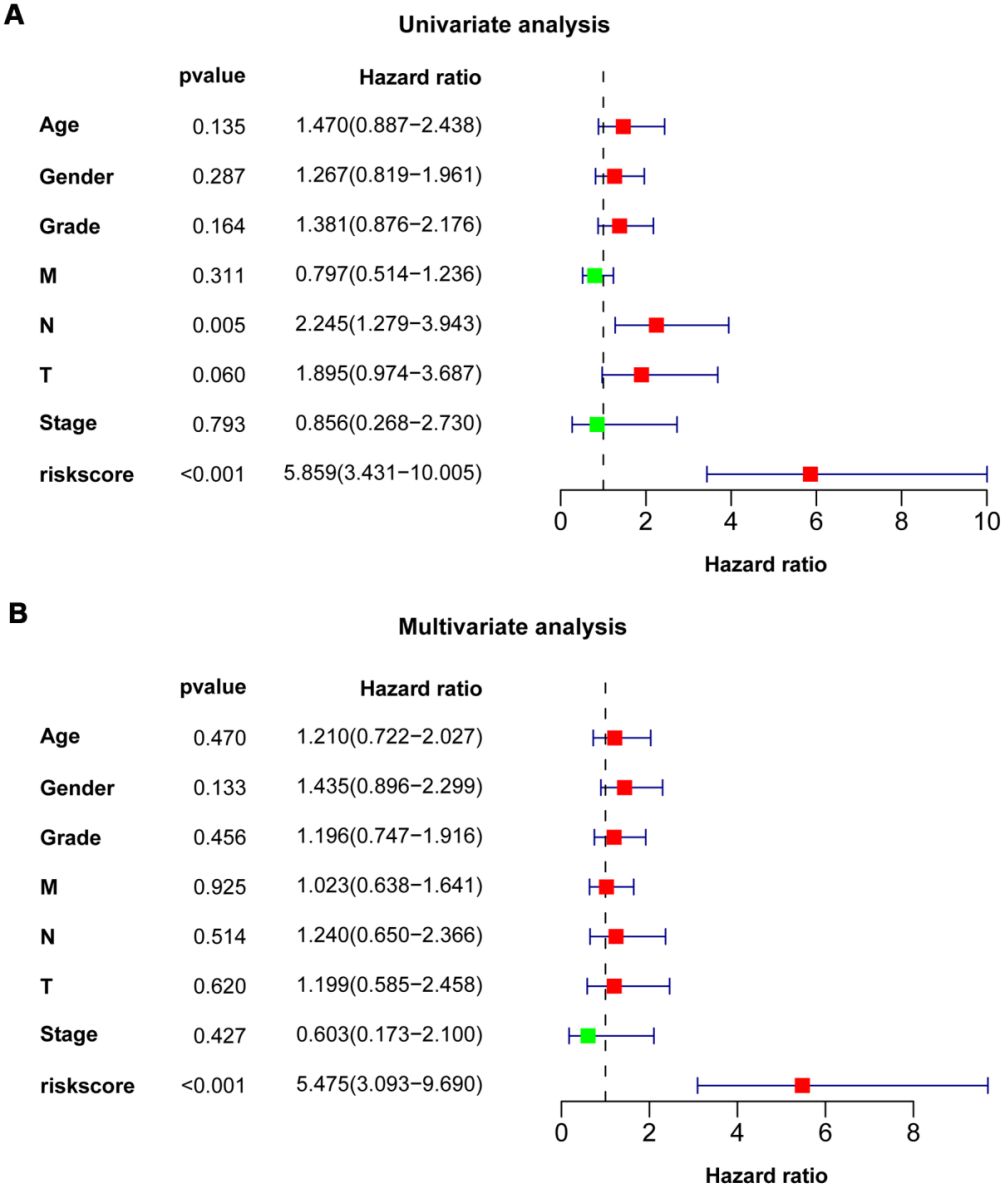
**Univariate and multivariate Cox regression analysis of clinicopathologic features.** Forest plots showed clinicopathologic features with prognostic significance in univariate (**A**) and multivariate (**B**) Cox regression analysis, respectively.

Univariate Cox regression analysis showed that N stage (HR = 2.245, *P* = 0.005) and eight-IRlncRNA signature (HR = 5.859, *P* < 0.001) were dramatically associated with the prognosis of PAAD patients (Figure 4A). Moreover, multivariate Cox regression analysis reflected that the eight-IRlncRNA signature (HR = 5.475, *P* <0.001) was significantly correlated with the prognosis of PAAD patients (Figure 4B). The results illustrated that the eight-IRlncRNA signature was a promising independent prognostic biomarker in PAAD.

### Nomogram development

**Figure 5:**
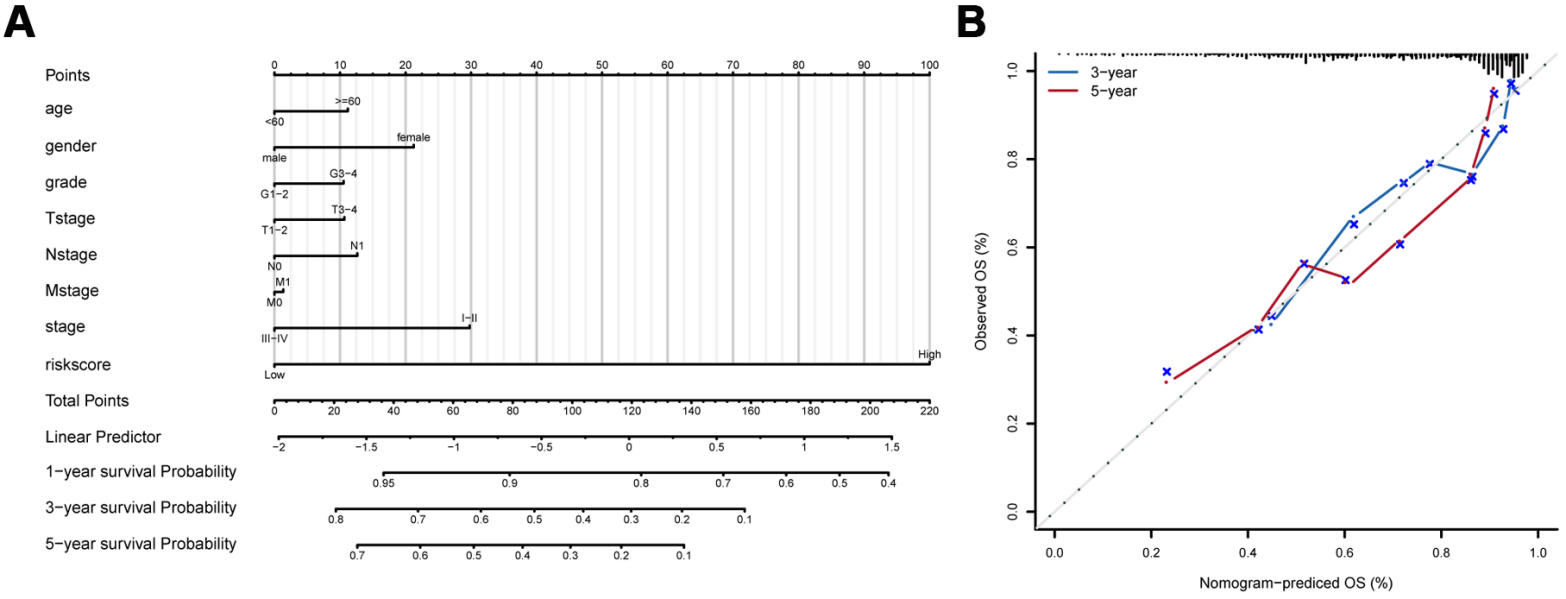
**Development of prognostic model based on eight-IRlncRNA signature and clinicopathologic features.** (**A**) Nomogram for predicting the survival probability of 1-, 3-, and 5-year overall survival for PAAD patients. (**B**) Prediction made by Calibration plot of the nomogram for overall survival.

To develop a clinical utility for predicting the survival probability of PAAD patients, we constructed a nomogram which was integrated with both the eight-IRlncRNA signature and clinicopathological features, including age, gender, grade, T stage, N stage, M stage, and AJCC stage (Figure 5A). The calibration plots displayed a good performance in 1-, 3-, and 5-year overall survival of the nomogram, which indicated that the nomogram had good accuracy as an ideal model (Figure 5B). The nomogram predicted the probability of 3- and 5-year OS in PAAD patients, providing a quantitative method to predict the OS of PAAD patients, and helped clinicians to make medical decisions and follow-up plans.

### GSEA and GSVA between high- and low-risk groups

**Figure 6:**
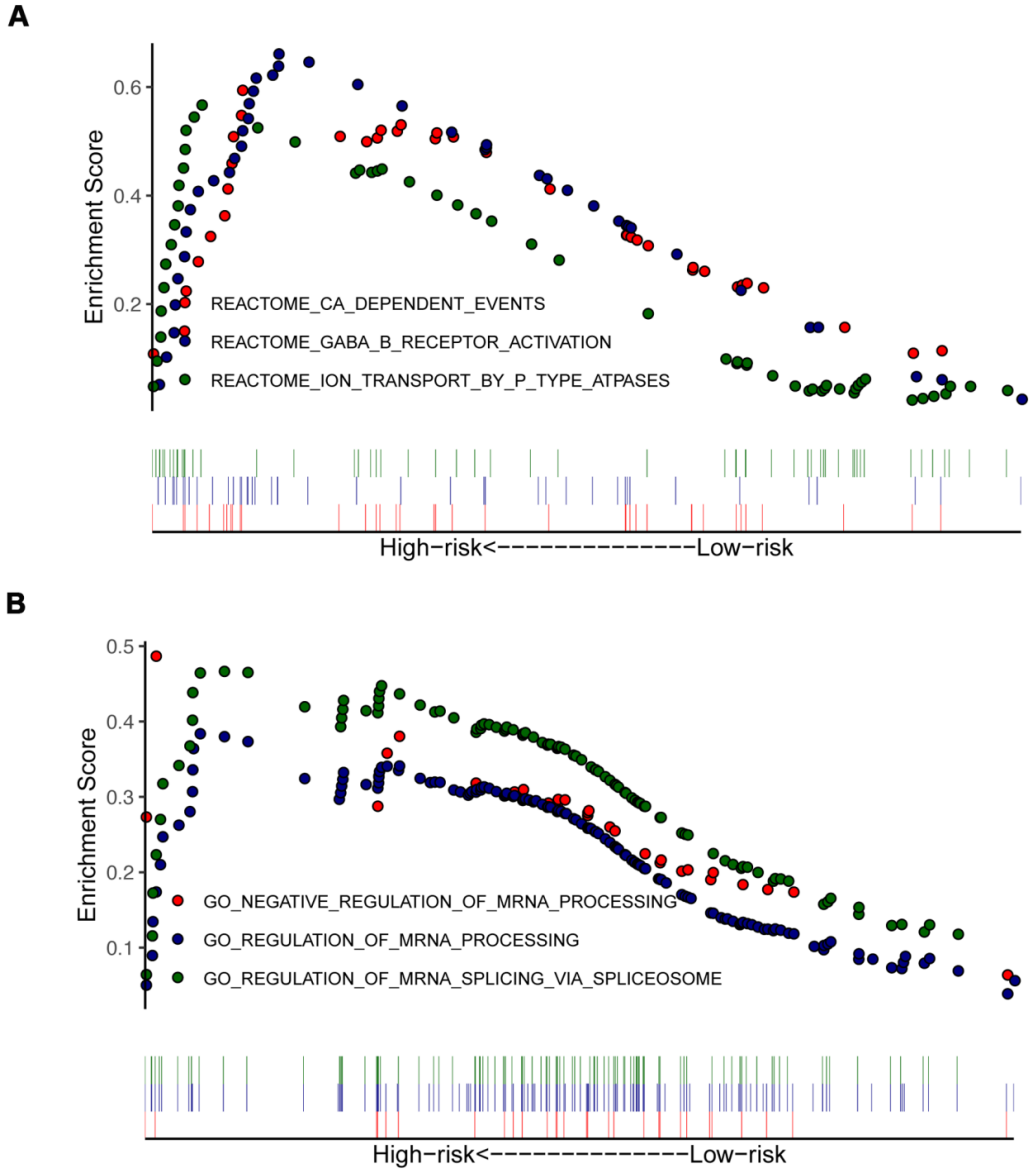
**Enrichment plots from gene set enrichment analysis (GSEA) in high- and low-risk groups.** GSEA indicated significant enrichment of immune-related phenotype in the high-risk patients, which were based on c2.all.v7.0.symbols.gmt (**A**) and c5.all.v7.0.symbols.gmt (**B**).

To identify the significant changes of functional phenotypes in high- and low-risk groups, we conducted GSEA and GSVA analyses between groups. The results of GSEA demonstrated that several signaling pathways were positively enriched in the high-risk group, including Reactome_GABA_B_Receptor_Activation (Figure 6A) and Regulation_of_mRNA_Processing (Figure 6B). For PAAD patients with high-risk score, GSVA uncovered that some signaling pathways were activated, including E2F_Targets, G2M_Checkpoint, and Glycolysis. Otherwise, Angiogenesis, Hedgehog_Signaling, and Myogenesis were inhibited (Supplementary Figure 4 and Supplementary Table 2).

### Correlation of eight-IRlncRNA signature with immune infiltration

**Figure 7:**
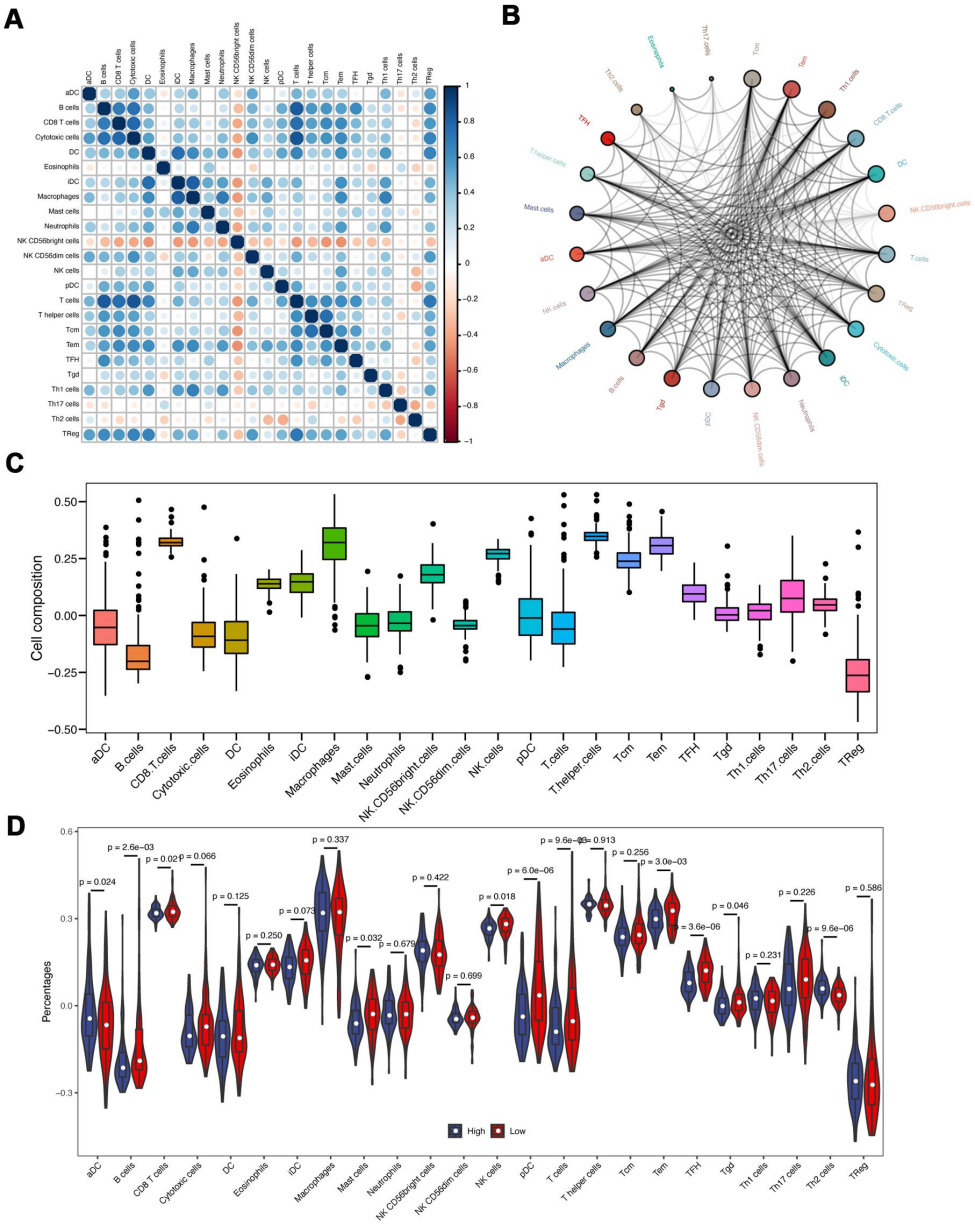
**Immune infiltration analyses.** (**A**) The correlation of different immune cells. (**B**) The immune cell interaction network. The big circle represented a strong interaction, and small one represented a weak interaction. (**C**) The composition analysis of immune cells in PAAD patients. The x-axis represents the type of immune cells, and the y-axis represents the proportion of immune cells. (**D**) The discrepancy of immune infiltration between high- and low-risk groups.

Since the functional enrichment analysis uncovered that IRlncRNA-targeted mRNAs contributed to an immune response of PAAD, we investigated the correlation of eight-IRlncRNA signature with immune cell infiltration in the tumor microenvironment. The correlation analysis between immune cells showed that T cells was negatively correlated with cytotoxic cells and B cells, however NK CD56 bright cells were positively related with other immune cells (Figure 7A).

The immune cell interaction network exhibited that Tcm, Tem, and Th1 cells had a strong relationship with other immune cells, but Th2 cells, Eosinophils, and Th17 cells had a weak relationship with other immune cells (Figure 7B). The composition analysis showed that CD8 T cells, T helper cells, and macrophages comprised the majority of immune cells in PAAD patients. By contrast, the minority of immune cells mainly included B cells and TReg (Figure 7C). Subsequently, we compared the differences of immune infiltration between high- and low-risk groups. The infiltration levels of aDC, B cells, CD8 T cells, Mast cells, NK cells, pDC, T cells, Tem, TFH and Th2 cells were significantly different (*P* < 0.05, Figure 7D).

**Figure 8:**
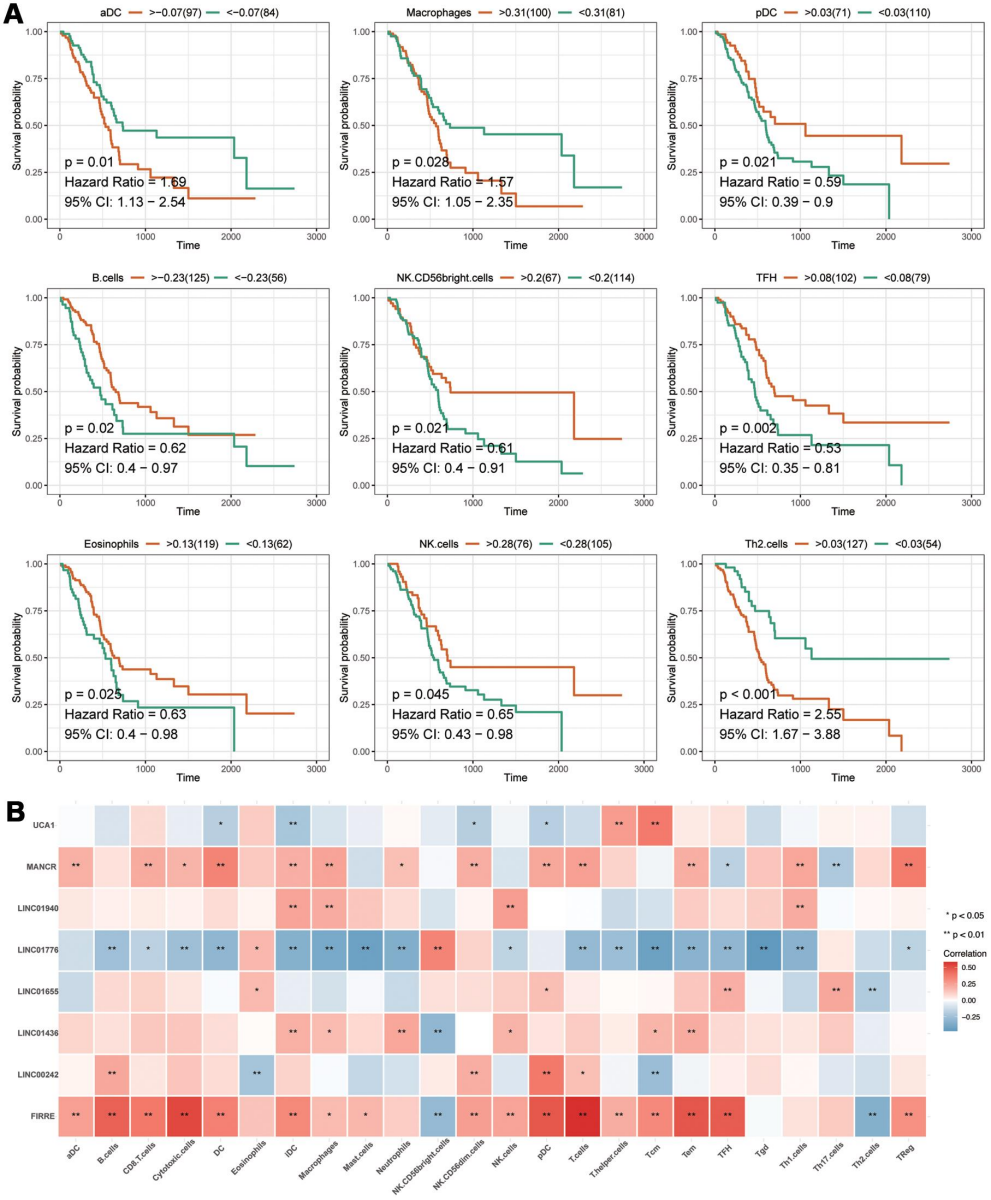
**The analyses of immune cell.** (**A**) The prognostic analysis of immune cells. The X-axis represents the survival time (day). Orange and green curve denote the high- and low-level groups, respectively. (**B**) The correlation of eight-IRlncRNA signature with immune cells. The X-axis represents the type of immune cells, and the Y-axis represents eight lncRNAs. Red means positive correlation, while blue means negative correlation. The darker color stands for a stronger correlation.

The prognostic analysis of immune cells showed that aDC, Macrophages, pDC, B cells, NK CD56bright cells, TFH, Eosinophils, NK cells and Th2 cells were related to the prognosis of patients with PAAD (Figure 8A). According to a correlation analysis of the eight IRlncRNAs and immune cells, we found that there were solid correlations of lncRNA FIRRE with T cells and cytotoxic cells. However, we observed a lack of correlation between the majority of immune cells and some lncRNA, including LINC01940 and LINC01655 (Figure 8B). Taken together, these findings illustrated that eight IRlncRNAs of the current signature involved in the process of pancreatic adenocarcinoma via mainly promoting immune response.

## DISCUSSION

This comprehensive analysis of PAAD generated a lncRNA-based signature which might be enabled to predict the prognosis for patients with PAAD. The data from three distinct cohorts, including one training cohort and two validation cohorts, offered advantages over a single cohort study. Following the identification of 132 among 1,471 IRlncRNAs, eight IRlncRNAs with prognostic value were served as a signature via an iterative Lasso Cox regression. Despite some members in this signature needed further investigation, the signature as a whole enhanced our understanding of the IRlncRNA-based biomarker to predict a prognosis for PAAD.

Based on multiple Lasso runs, iterative Lasso regression was implemented to screen the consensus genes and finalize a signature of eight IRlncRNAs for prognostic prediction of PAAD patients. Iterative Lasso regression, a procedure running Lasso in a loop, has an advantage in constructing the significant and independent predictive model [12]. According to the risk score in each sample, eight-IRlncRNA signature divided PAAD samples into high- and low-risk groups, and samples in high-risk group showed worse OS and RFS than those in low-risk group. In order to assess the robustness of eight-IRlncRNA signature, we used two other independent cohorts (PACA-CA and PACA-AU) as validation sets. The signature demonstrated an accurate prediction in at 1-, 3-, 5-year survival probability, and in other respects (OS, risk score and RFS) also showed prognostic power. An understanding of how lncRNA signature contributes the prognosis in cancer could enable its use as a prognostic model [13]. Moreover, previous reports indicated that MUC1, COPS6, HOTAIR, COL6A1 were served as biomarkers for PAAD and had tumor-suppressive or tumor-promoting ability [9, 14–16]. Here, we compared the signature to known prognostic models, illustrating that eight-IRlncRNA had a good performance for survival prediction. Therefore, the result from the comparison indicated that eight-IRlncRNA signature could be a valid prognostic predictor for patients with PAAD.

Many factors, such as pathological grade, TNM stages, were thought to be major prognostic factors of pancreatic adenocarcinoma. The instable efficacy of PAAD treatment attributed to patients with clinicopathologic features, including age, gender, history of alcohol exposure, pathological grade, TNM stages, and AJCC stage. In this study, PAAD patients were stratified into subgroups according to different clinicopathologic characteristics. As expected, the predictive ability of eight-IRlncRNA signature was effective in all subgroups. Combined with the signature and the clinicopathologic characteristics, a nomogram was built to predict the likelihood of survival probability in patients with PAAD. The predicted outcome was available to support the clinicians for designing an individualized treatment. All of these results revealed that the eight-IRlncRNA signature, an independent prognostic biomarker, could provide accurate prognosis of PAAD patients with different clinicopathologic features, reflecting a wide applicability for prognostic prediction.

Here, the prognostic signature consisted of eight lncRNAs. Of those, three lncRNAs had not even been reported, including LINC01665, LINC01940, and LINC01776. The detailed mechanism of these lncRNAs has yet to be fully studied. Some evidence showed that UCA1 acted as a sponge by targeting miR-193a-3p [17], miR-204-5p [18] and miR-135a [19]. It was also observed that aberrant expression of UCA1 facilitated to immune escape of gastric cancer [17], which was consistent with our findings that UCA1 might be involved in the immune response of cancer. In pancreatic adenocarcinoma, UCA1 overexpression was found to be associated with shorter overall survival [20], which enabled UCA1 as a prognostic marker. Its application in clinical management, however, remained to be studied further.

Similar to UCA1, MANCR is available to promote cell proliferation, viability, and genomic stability by sponging miRNAs, including miR-218, miR-101, and miR-122a [21, 22]. Whereas, MANCR can also interact with protein to promote the migration and invasion of prostate cancer [23]. A study on gastric cancer demonstrated that high expression of MANCR predicted poor survival in patients [24]. FIRRE is a lncRNA binding with protein to increase inflammatory genes expression in the innate immune system [25]. It has previously been observed that higher expression of FIRRE was significantly related to longer overall survival of patients with colorectal cancer, but single FIRRE was not an independent predictor for survival probability of patients [26].

LINC01436 and LINC00242 appear to render cancer cell to progression. It has been reported that LINC01436 can promote cell growth of non-small cell lung cancer and gastric cancer [27, 28]. In accordance with the present result in PAAD, LINC01436 was associated with poor overall survival of two above cancers. In gastric cancer, LINC00242 was also found to drive tumorigenesis and contributed to poor prognosis [29]. In this study, it is somewhat surprising that high expression of LINC00242 was found in the low-risk group of PAAD patients. The discrepancy of LINC00242 level may arise from different cancer types. The molecular event implicated with LINC00242 remains further investigation in future studies.

In the initial stage of this study, there were identical GO items observed in the functions of IRGs and IRlncRNA-targeted genes, including immune effector process, innate immune response, production of molecular mediator of immune response and so on. Although the immune infiltration scenario can hardly be exemplified by the recent studies of eight lncRNAs, it was speculated from our findings that these eight lncRNAs might influence the progression of PAAD when subjected to immune infiltration. Accordingly, our data on function enrichment add to the growing list of participants in tumor immune response.

Given the potential role of IRlncRNA in the process of immune infiltration, the mechanism underlying that each IRlncRNA member in the current signature regulated immune response of PAAD should be explored in further studies. In addition, although a total of 435 PAAD samples with prognostic information was enrolled in this study, it was worth to investigate the general applicability of this prognostic signature by prospective studies with more clinical samples. The signature in this study clearly has potential as a prognostic model in patients with PAAD. Further studies need to be done, however, to firmly establish its position as a predicted model in this cancer.

In summary, the robust statistical power provided by three distinct cohorts of clinically annotated samples enabled us to establish an eight-IRlncRNA signature, which facilitated a prognosis prediction in patients with PAAD. According to the immunoregulatory potential of eight IRlncRNAs in PAAD, a better understanding of immune-related molecules may entail a further investigation to explore detailed mechanism and provide a rationale for immune therapy against PAAD. Importantly, the signature described here may contribute to advancing the clinical management of PAAD patients.

## MATERIALS AND METHODS

### PAAD cohorts acquisition and prognostic immune-related genes (IRGs) identification

TCGA-PAAD data and the corresponding clinical information were downloaded from UCSC Xena (https://xenabrowser.net/datapages/). PAAD samples without prognostic information were excluded. A total of 181 PAAD samples were enrolled as a training cohort. Both 164 PAAD samples from Pancreatic Cancer-CA (PACA-CA) and 90 PAAD samples from Pancreatic Cancer-AU (PACA-AU) were downloaded from ICGC (https://dcc.icgc.org/) respectively, serving as the validation cohorts. The exclusion criterion was samples without survival information. Our study was in accordance with the publication guidelines provided by TCGA. This study does not contain any studies with human participants or animals performed by any of authors.

First, data pre-processing was performed on the gene expression profiles and corresponding clinical information. Then, IRGs were collected from ImmPort (https://immport.niaid.nih.gov) [30]. Second, based on PAAD data from TCGA, prognostic IRGs were screened using univariate Cox regression analysis. The *P*-value < 0.05 was considered significant. Third, Gene ontology (GO) and Kyoto Encyclopedia of Genes and Genomes (KEGG) pathway enrichment analyses were applied to analyze the biological function of prognostic IRGs.

### IRlncRNAs identification and functional enrichment analyses

The expression matrixes of mRNA and lncRNA were distinguished based on classification and annotation analyses. IRlncRNAs were identified according to |Pearson coefficient| > 0.4 and *P* < 0.01. To investigate possible functions of IRlncRNAs during the progression of PAAD, IRlncRNAs-targeted genes were collected using co-expression analysis according to |Pearson coefficient| > 0.4 and *P* < 0.01. Next, the clusterProfiler package in the R software was applied to perform GO and KEGG pathway enrichment analyses for IRlncRNAs-targeted genes [31]. Adj. *P* < 0.05 was considered significant.

### Construction and assessment of the prognostic signature

Iterative Lasso regression was performed to screen the consensus genes based on multiple times of Lasso operation. The consensus genes were further incorporated into Cox model. The process of incorporation was stopped as the area under curve (AUC) of ROC reached a peak [32]. To identify the optimal prognostic signature of IRlncRNAs, univariate Cox regression analysis was carried out to screen prognostic IRlncRNAs. The *P*-value < 0.01 was set as the criteria of univariate Cox regression. Subsequently, iterative Lasso Cox regression was utilized by the glmnet package in the R software to construct the optimal prognostic signature of IRlncRNAs. The consensus genes were identified according to the frequency > 100 within 1000 times of Lasso Cox regression.

The prognostic signature for PAAD patients was constructed based on the consensus gene expression. Following this, a prognostic risk score was calculated for each sample according to this signature, and PAAD patients were divided into high- and low-risk groups based on the median value of the risk score. Finally, the R software was utilized to draw a correlation diagram of risk factors, which showed the prognosis of the high- and low-risk groups.

### Comparison of the signature with other prognostic biomarkers in two validation cohorts

The fact that a signature predicts the prognosis for PAAD patients pleas for an advantage of this signature when compared to other biomarkers in independent cohorts. To further the understanding of the predictive power of the signature, we conducted ROC analysis to compare a prognostic performance of the signature with those of four prognostic models reported in previous studies, including MUC1 model, COPS6 model, HOTAIR model, and COL6A1 model. These prognostic models were compared with the present signature in two independent validation cohorts to validate the potential of the signature.

### Survival analysis of subgroups and nomogram construction

A good prognostic signature may enable its use as an independent biomarker from clinicopathologic prognostic characteristics. The PAAD patients were divided into subgroups followed by Kaplan-Meier survival analysis for each subgroup. Univariate and multivariate Cox regression were utilized to assess the independence and applicability of the signature. The signature and clinicopathologic features were integrated to construct a nomogram, which was applied to predict PAAD patients’ prognosis.

### Gene set enrichment analysis (GSEA) and Gene Set Variation Analysis (GSVA)

GSEA and GSVA were performed to analyze the important functional phenotypes between high- and low-risk groups. Two gene sets, including c2.all.v7.0.symbols.gmt and c5.all.v7.0.symbols.gmt, were utilized as the reference gene sets. GSEA 4.0.3 software was used to perform a standard enrichment analysis with 1000 permutations [33], in order to explore biological function of prognostic signature. A nominal *P* < 0.05 and a false discovery rate < 0.05 were statistically significant. GSVA was performed with the clusterProfiler package and the GSVA package [34]. The gene set of h.all.v7.1.symbols.gmt. was set as the reference gene set. The adj. *P* < 0.05 was considered significant.

### Immune infiltration, prognostic analysis and correlation analysis of the signature

The immune infiltration was analyzed by GSVA package to investigate the composition of immune cell infiltration, differences between high- and low- risk groups, and cell-cell interaction. Kaplan-Meier survival curves were generated to assess overall survival (OS) in high- and low-risk groups. The correlation analysis was conducted between the present signature and immune cell infiltration, which was visualized by using ggplot2 package.

## Supplementary Material

Supplementary Figures

Supplementary Table 1

Supplementary Table 2
