# Supplementary material for: An immune-related lncRNA signature for the prognosis of pancreatic adenocarcinoma
Source: Aging (Albany NY). 2021 Jul 20;13(14):18806–26. doi: 10.18632/aging.203323 (PMC8351726; doi:10.18632/aging.203323)
Supplement: Supplementary Figures [file aging-13-203323-s001.pdf]

SUPPLEMENTARY FIGURES

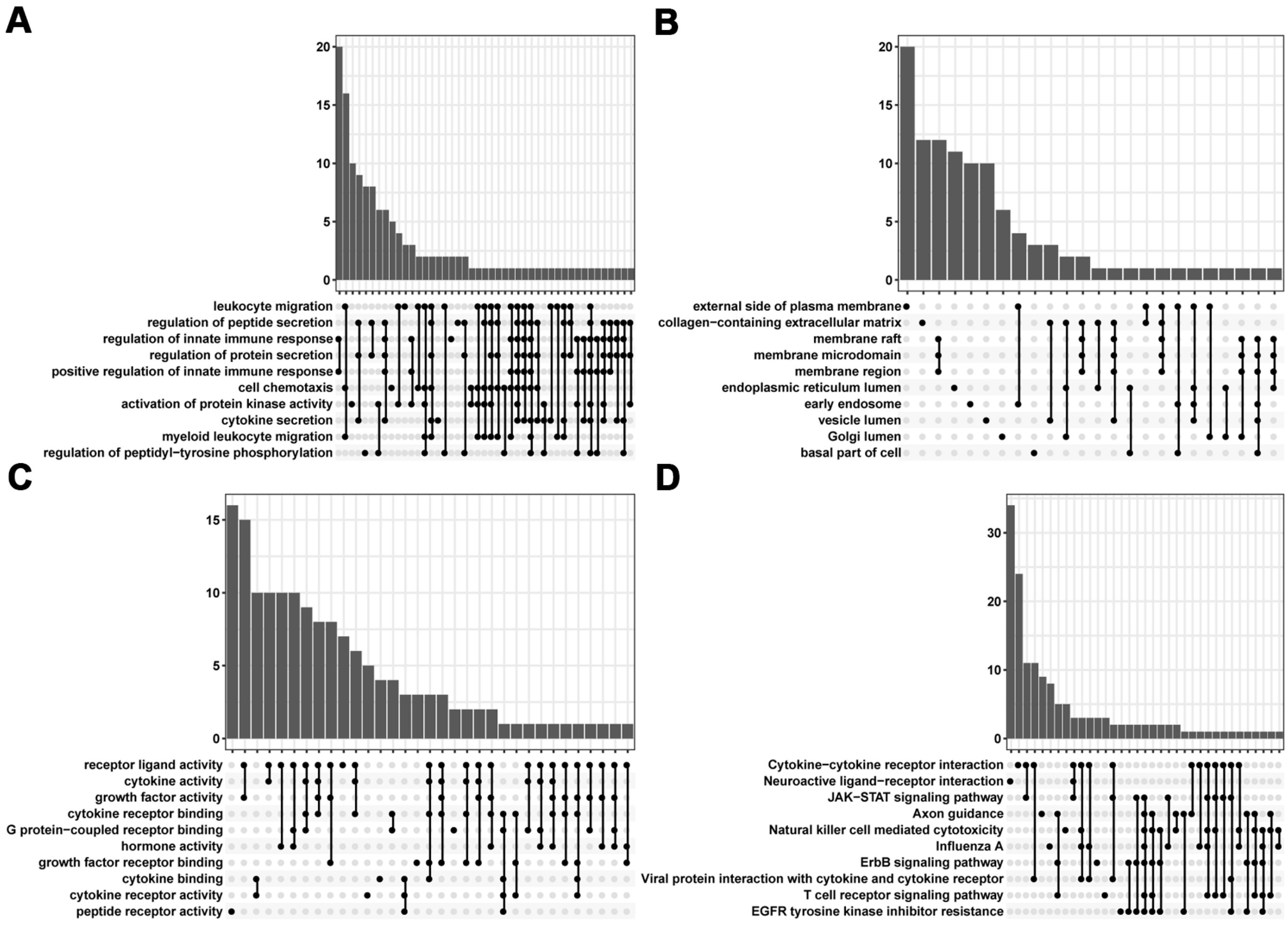

Supplementary Figure 1. Gene ontology (GO) and Kyoto Encyclopedia of Genes and Genomes (KEGG) enrichment analyses for prognostic IRGs. Biological process (BP) (**A**); Cellular component (CC) (**B**); Molecular function (MF) (**C**); KEGG signaling pathways (**D**).

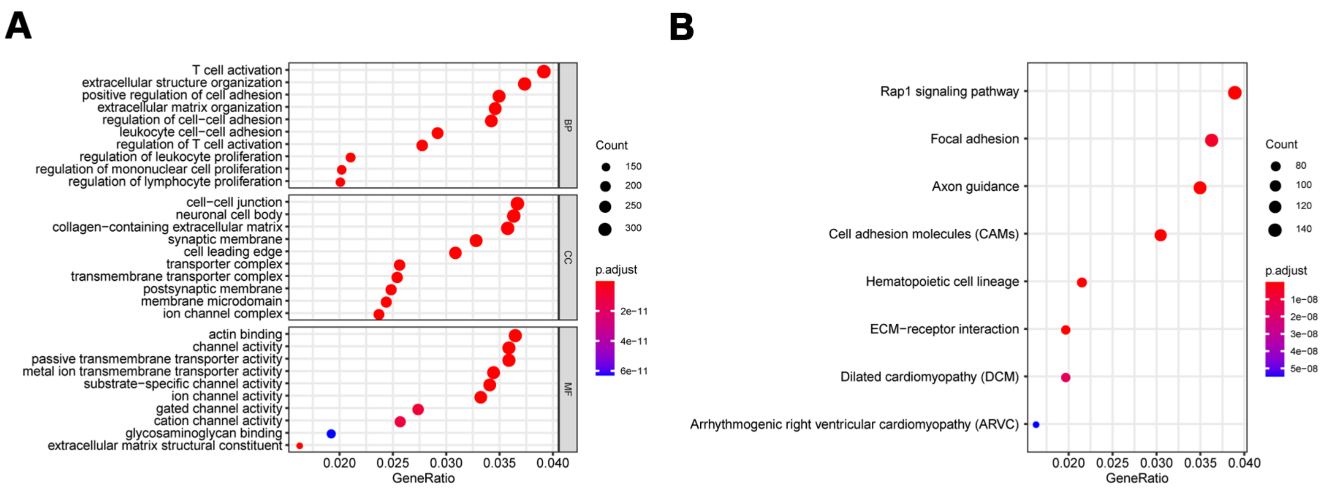

Supplementary Figure 2. Gene ontology (GO) and Kyoto Encyclopedia of Genes and Genomes (KEGG) enrichment analyses for lncRNA-targeted genes. (**A**) GO analysis including Biological process (BP), Cellular component (CC), and Molecular function (MF). (**B**) KEGG analysis including the main pathways. The X-axis indicates the gene ratio between the number of enriched genes and the total number of genes in GO terms, the Y-axis represents GO terms. The color represents the adjusted *P* value, and the point size denotes the number of genes.

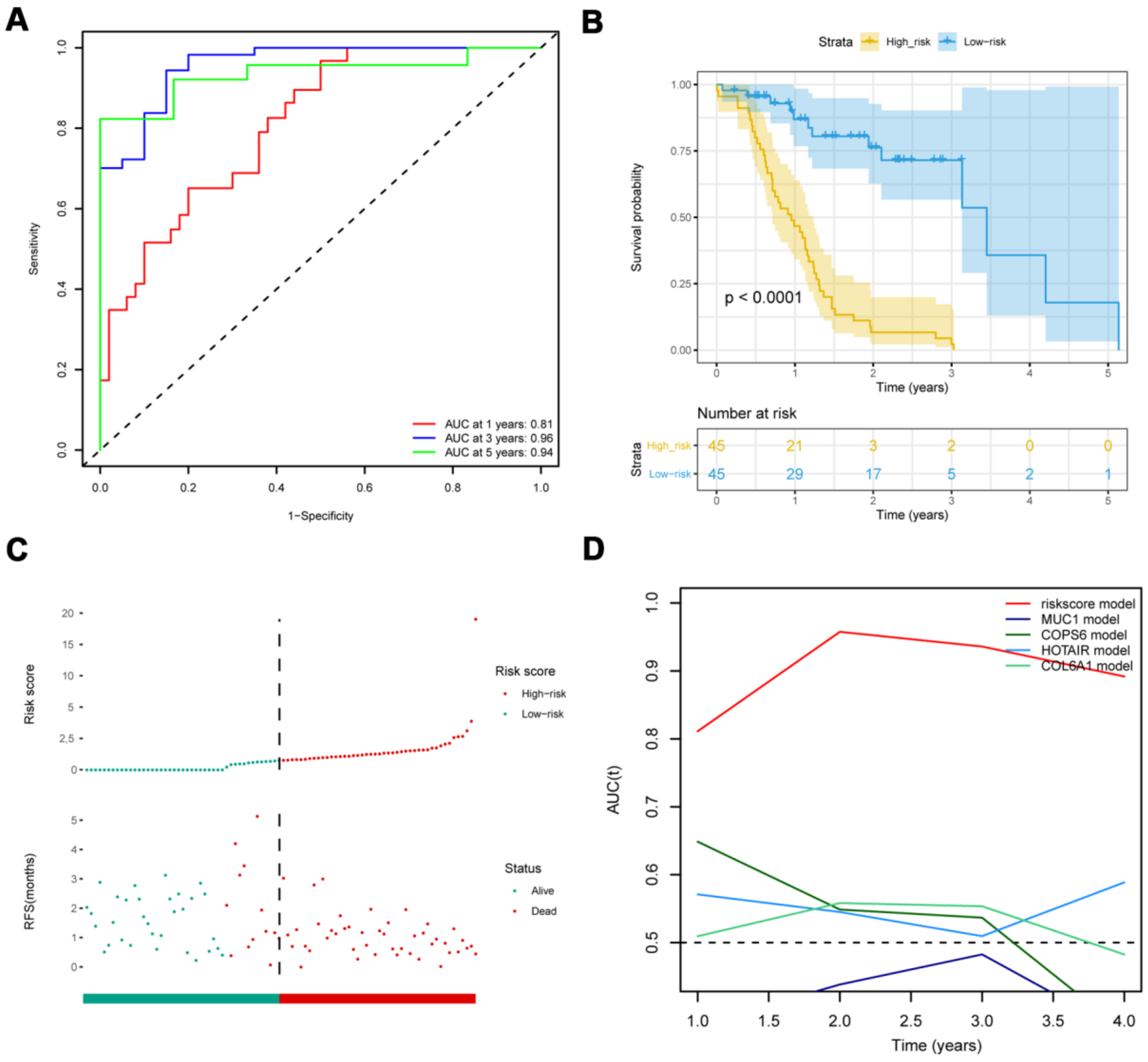

Supplementary Figure 3. Comparison with other prognosis biomarkers and validation of the eight-IRlncRNA signature in the validation cohort (PACA-AU). (**A**) ROC curve of eight-IRlncRNA signature for 1, 3, 5- year survival. (**B**) Kaplan-Meier curves for the high- and low-risk groups. (**C**) Risk score distribution and the survival status for patients. (**D**) Time-dependent ROC curve of the eight-IRlncRNA signature compared with other prognosis biomarkers.

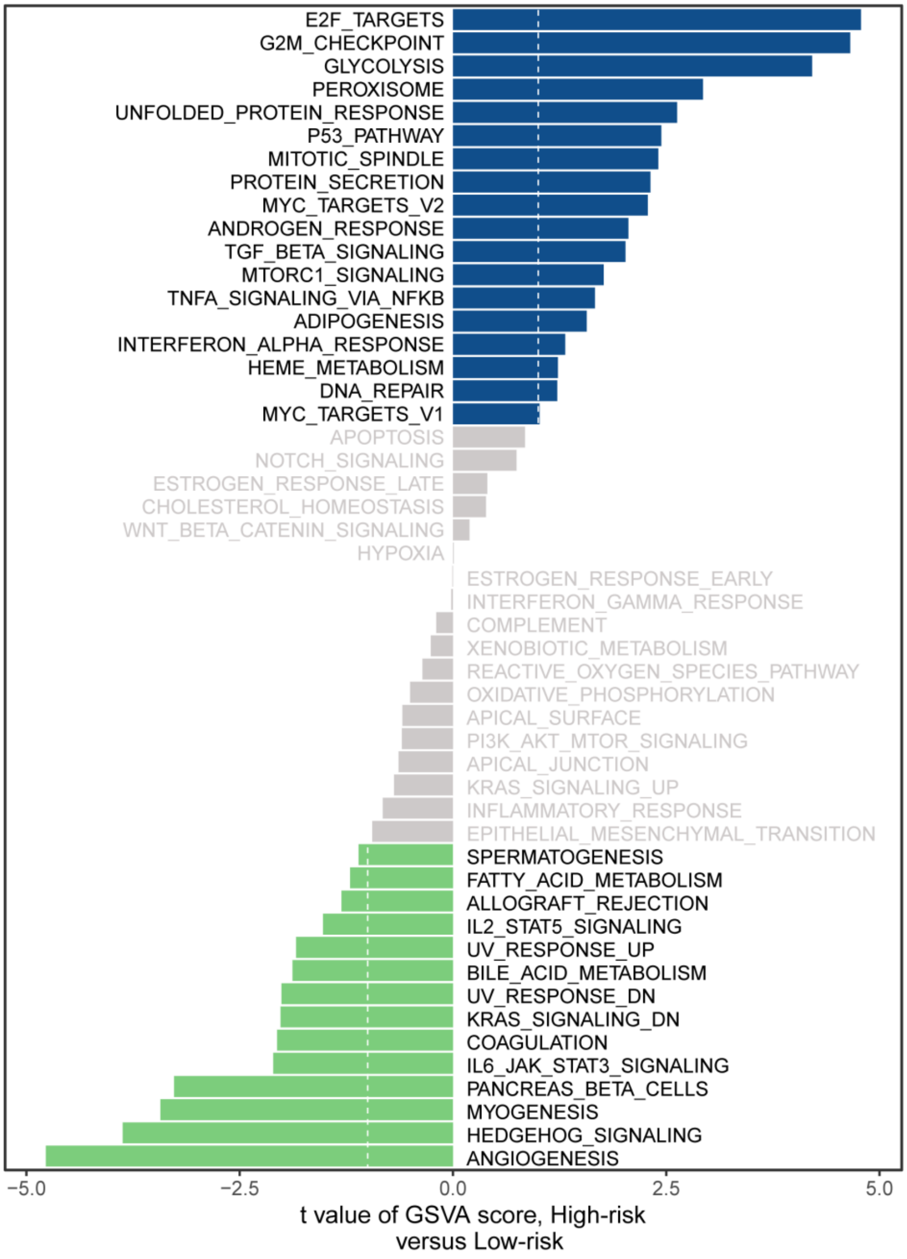

Supplementary Figure 4. Gene set variation analysis (GSVA) of high- and low-risk groups revealed significant difference in enrichment of h.all.v7.0.symbols.gmt.
