# Supplementary material for: An immune-related lncRNA signature for the prognosis of pancreatic adenocarcinoma
Source: Aging (Albany NY). 2021 Jul 20;13(14):18806–26. doi: 10.18632/aging.203323 (PMC8351726; doi:10.18632/aging.203323)
Supplement: Supplementary Table 1 [file aging-13-203323-s002.doc]

Supplementary Table 1. The identification of 132 prognostic IRlncRNAs among 1,471 IRlncRNAs.

| **gene** | **HR** | **z** | **pvalue** | **lower** | **upper** |
| --- | --- | --- | --- | --- | --- |
| **CABIN1** | 0.36240737 | -3.2335331 | 0.00122269 | 0.195889 | 0.67047719 |
| **PHF1** | 0.58199024 | -2.6448332 | 0.00817312 | 0.38967771 | 0.86921226 |
| **MAPK4** | 0.86212336 | -2.7896313 | 0.00527681 | 0.77678566 | 0.95683627 |
| **PART1** | 0.83498345 | -3.3888077 | 0.00070197 | 0.75227941 | 0.9267798 |
| **TUBA3FP** | 0.61273256 | -3.7460674 | 0.00017963 | 0.47420912 | 0.79172073 |
| **ZNF667-AS1** | 0.76443535 | -2.755383 | 0.00586235 | 0.6314784 | 0.92538621 |
| **CCDC13-AS1** | 0.70374592 | -3.435786 | 0.00059084 | 0.57593491 | 0.85992065 |
| **MIR4435-2HG** | 1.55236731 | 3.05870169 | 0.00222298 | 1.17113907 | 2.05769266 |
| **C8orf31** | 1.23645968 | 3.93067856 | 8.47E-05 | 1.11228489 | 1.37449727 |
| **LINC00174** | 0.73628163 | -2.6386915 | 0.00832267 | 0.58652571 | 0.92427431 |
| **LINC00476** | 0.54925504 | -3.5401992 | 0.00039983 | 0.39418936 | 0.76532024 |
| **C9orf139** | 0.65802794 | -3.5745136 | 0.00035088 | 0.52310003 | 0.82775902 |
| **LINC00334** | 0.72693604 | -3.3465974 | 0.0008181 | 0.60308738 | 0.87621798 |
| **LINC01559** | 1.20485057 | 4.04272959 | 5.28E-05 | 1.10076802 | 1.3187746 |
| **OGFRP1** | 1.34519556 | 2.60551478 | 0.00917363 | 1.07623793 | 1.68136714 |
| **CYP51A1-AS1** | 0.71195867 | -3.5364787 | 0.0004055 | 0.58977077 | 0.8594613 |
| **GOLGA8M** | 0.74320394 | -2.9300986 | 0.00338854 | 0.60938424 | 0.9064102 |
| **LINC00158** | 0.78668293 | -2.710663 | 0.00671488 | 0.66138902 | 0.93571258 |
| **LINC00265** | 0.69633165 | -2.9923874 | 0.00276805 | 0.54936795 | 0.88261022 |
| **C9orf163** | 0.82952421 | -2.6159786 | 0.00889721 | 0.72112997 | 0.95421138 |
| **FLVCR1-DT** | 0.69309687 | -4.0703461 | 4.69E-05 | 0.58094178 | 0.82690434 |
| **RAMP2-AS1** | 0.7042718 | -3.5031273 | 0.00045983 | 0.57883203 | 0.85689587 |
| **SCOC-AS1** | 0.61188826 | -3.9627406 | 7.41E-05 | 0.47991083 | 0.78016001 |
| **ARRDC1-AS1** | 0.65625538 | -2.7731897 | 0.00555097 | 0.48729093 | 0.88380697 |
| **ATP6V0E2-AS1** | 0.74868203 | -3.4621609 | 0.00053586 | 0.63552979 | 0.88198034 |
| **FAM83A-AS1** | 1.19625613 | 4.33068673 | 1.49E-05 | 1.1030694 | 1.29731522 |
| **MIR29B2CHG** | 0.73088119 | -3.3493098 | 0.00081013 | 0.60837595 | 0.87805461 |
| **ZNRD1ASP** | 0.68094514 | -2.6316513 | 0.0084971 | 0.5114695 | 0.90657661 |
| **H1FX-AS1** | 0.70374196 | -3.2333761 | 0.00122336 | 0.56874894 | 0.87077568 |
| **HCP5** | 1.41813406 | 3.0710628 | 0.00213298 | 1.13472529 | 1.77232693 |
| **RFPL3S** | 0.75018015 | -2.6027296 | 0.00924849 | 0.60417123 | 0.93147479 |
| **SRRM2-AS1** | 0.67530521 | -2.8779942 | 0.00400212 | 0.51687641 | 0.88229433 |
| **FIRRE** | 1.23958168 | 3.24201901 | 0.00118686 | 1.08864334 | 1.41144735 |
| **LINC01089** | 0.67307477 | -3.9885366 | 6.65E-05 | 0.55408162 | 0.81762258 |
| **ALOX12-AS1** | 0.54237746 | -2.9107187 | 0.00360599 | 0.35924502 | 0.81886538 |
| **HLA-F-AS1** | 1.31054475 | 2.79246914 | 0.00523075 | 1.08396522 | 1.58448585 |
| **LINC00612** | 0.77989205 | -2.7164096 | 0.00659942 | 0.65183011 | 0.9331137 |
| **LINC00887** | 1.23315977 | 2.91519596 | 0.00355465 | 1.07108667 | 1.41975721 |
| **PAXIP1-AS2** | 1.62172147 | 2.88037698 | 0.003972 | 1.16707047 | 2.25348905 |
| **POLR2J4** | 0.58604295 | -3.1671899 | 0.0015392 | 0.42103322 | 0.81572268 |
| **UCA1** | 1.22327184 | 4.77993191 | 1.75E-06 | 1.12625062 | 1.32865099 |
| **CYTOR** | 1.40030469 | 3.00686815 | 0.00263954 | 1.12437309 | 1.74395246 |
| **CLDN10-AS1** | 1.23551323 | 4.05598608 | 4.99E-05 | 1.11548622 | 1.36845521 |
| **HCG14** | 0.76231978 | -2.8268387 | 0.004701 | 0.63156497 | 0.92014515 |
| **LGALSL-DT** | 0.73863299 | -2.6007453 | 0.00930215 | 0.5878607 | 0.92807478 |
| **LINC00885** | 1.17732972 | 3.02606334 | 0.0024776 | 1.0591996 | 1.30863463 |
| **LINC01133** | 1.1809968 | 3.28182971 | 0.00103136 | 1.06930246 | 1.3043582 |
| **LINC01142** | 1.36943321 | 2.82451191 | 0.00473527 | 1.10101556 | 1.70328865 |
| **LINC01527** | 1.31653887 | 2.65599096 | 0.00790757 | 1.07472834 | 1.61275602 |
| **LINP1** | 1.1718443 | 3.04689335 | 0.0023122 | 1.05820111 | 1.29769195 |
| **MEG9** | 0.80590454 | -3.0807505 | 0.0020648 | 0.70252457 | 0.92449738 |
| **POU6F2-AS1** | 0.78306925 | -3.1298156 | 0.00174916 | 0.67188562 | 0.91265155 |
| **SCEL-AS1** | 1.60958417 | 2.79932511 | 0.00512095 | 1.15340514 | 2.24618489 |
| **SLC25A5-AS1** | 0.56987308 | -3.2232591 | 0.00126741 | 0.40483159 | 0.80219858 |
| **STK24-AS1** | 0.71527045 | -2.9035773 | 0.00368926 | 0.57047321 | 0.89682006 |
| **CYP4A22-AS1** | 1.277541 | 2.72480961 | 0.00643386 | 1.0711717 | 1.52366891 |
| **LAMP5-AS1** | 0.76717307 | -2.6105954 | 0.00903848 | 0.62874481 | 0.93607854 |
| **LINC00705** | 1.22834177 | 3.22966041 | 0.00123937 | 1.08421302 | 1.39163013 |
| **LINC00853** | 1.3441748 | 3.1364181 | 0.00171025 | 1.11733418 | 1.61706848 |
| **LINC01534** | 0.63463189 | -3.5713877 | 0.00035509 | 0.49447763 | 0.81451132 |
| **LINC01671** | 1.19553112 | 2.62419296 | 0.00868546 | 1.04624167 | 1.36612286 |
| **LINC01776** | 1.37409605 | 4.45731008 | 8.30E-06 | 1.19489117 | 1.58017733 |
| **NAALADL2-AS1** | 2.19395261 | 3.05750424 | 0.00223188 | 1.32583353 | 3.63049201 |
| **P3H2-AS1** | 1.27613857 | 3.69695726 | 0.0002182 | 1.12138687 | 1.45224606 |
| **UBXN7-AS1** | 0.59356345 | -3.6803343 | 0.00023293 | 0.44960068 | 0.7836233 |
| **BAIAP2-DT** | 0.52906075 | -3.0487044 | 0.00229831 | 0.35135944 | 0.79663516 |
| **BSN-DT** | 0.75533774 | -3.5604098 | 0.00037028 | 0.64723103 | 0.88150146 |
| **DANCR** | 0.58424303 | -3.1597799 | 0.00157888 | 0.41861523 | 0.81540254 |
| **DDC-AS1** | 0.56046099 | -2.6409284 | 0.00826792 | 0.36469386 | 0.86131564 |
| **MGAT3-AS1** | 1.25049172 | 2.69337283 | 0.00707331 | 1.06276008 | 1.4713853 |
| **MIS18A-AS1** | 0.7738072 | -2.7413783 | 0.0061182 | 0.64418429 | 0.92951286 |
| **NCAM1-AS1** | 0.66056202 | -3.5384746 | 0.00040245 | 0.52500519 | 0.83111975 |
| **PSPC1-AS2** | 0.75137568 | -2.5918313 | 0.00954666 | 0.605312 | 0.93268498 |
| **SGMS1-AS1** | 0.59076296 | -2.6505713 | 0.00803558 | 0.40029935 | 0.87184973 |
| **SLC16A1-AS1** | 1.40990672 | 2.92824221 | 0.00340884 | 1.12029552 | 1.77438625 |
| **SLC8A1-AS1** | 0.80115594 | -2.7873303 | 0.00531443 | 0.68551015 | 0.93631122 |
| **ZNF32-AS1** | 0.6894002 | -3.3951233 | 0.00068598 | 0.55618962 | 0.85451546 |
| **CCDC183-AS1** | 0.66049341 | -3.0769639 | 0.00209121 | 0.50714056 | 0.86021821 |
| **DNMBP-AS1** | 1.37553175 | 3.68768609 | 0.0002263 | 1.16111445 | 1.62954444 |
| **LAMA5-AS1** | 0.83460796 | -2.7339253 | 0.00625842 | 0.73315023 | 0.95010603 |
| **LINC00578** | 1.21513426 | 2.77078402 | 0.00559215 | 1.0586777 | 1.39471272 |
| **LINC01004** | 0.64579016 | -3.7294115 | 0.00019193 | 0.51319972 | 0.81263671 |
| **LINC01128** | 0.60548362 | -2.8803328 | 0.00397256 | 0.43035895 | 0.85187125 |
| **LINC01277** | 0.77810802 | -2.8157737 | 0.00486599 | 0.6534261 | 0.92658082 |
| **LINC01341** | 0.75854543 | -2.9677321 | 0.00300006 | 0.63200201 | 0.91042617 |
| **LINC01655** | 1.3834493 | 4.64385043 | 3.42E-06 | 1.20633768 | 1.58656403 |
| **LINC01740** | 0.65546749 | -2.6254375 | 0.00865377 | 0.47819096 | 0.89846456 |
| **LINC01757** | 3.1321689 | 3.22027304 | 0.00128069 | 1.56335465 | 6.2752761 |
| **LINC01940** | 1.22112511 | 4.57196716 | 4.83E-06 | 1.12089984 | 1.33031202 |
| **LINC02041** | 1.24273124 | 3.4577661 | 0.00054467 | 1.09870581 | 1.40563644 |
| **LINC02577** | 1.18436912 | 3.60459044 | 0.00031265 | 1.08026208 | 1.29850917 |
| **RNF144A-AS1** | 1.20873325 | 2.59187827 | 0.00954535 | 1.04730367 | 1.39504531 |
| **SNAP25-AS1** | 0.84644725 | -2.5789487 | 0.00991015 | 0.74572184 | 0.96077775 |
| **UBAC2-AS1** | 0.67030974 | -2.8193075 | 0.00481274 | 0.50757875 | 0.8852127 |
| **ALMS1-IT1** | 1.35249712 | 3.72306813 | 0.00019682 | 1.1537221 | 1.58551914 |
| **C9orf147** | 0.75617276 | -2.6733505 | 0.00750977 | 0.616073 | 0.9281323 |
| **DSCR9** | 0.72640229 | -3.3566968 | 0.0007888 | 0.60272477 | 0.87545811 |
| **ERVMER61-1** | 1.45980952 | 2.68464587 | 0.00726067 | 1.10751449 | 1.92416792 |
| **ITGB1-DT** | 1.26582404 | 3.33757616 | 0.00084513 | 1.1021874 | 1.45375505 |
| **LINC00242** | 0.64818897 | -3.4331087 | 0.0005967 | 0.50605997 | 0.83023549 |
| **LINC01284** | 0.75659063 | -2.746255 | 0.00602799 | 0.62002001 | 0.92324339 |
| **MIR5689HG** | 1.45586694 | 3.6733628 | 0.00023938 | 1.19147868 | 1.77892275 |
| **MYCBP2-AS2** | 0.65082743 | -2.8216568 | 0.00477763 | 0.4829437 | 0.87707189 |
| **MYOSLID** | 1.26267725 | 3.13594771 | 0.001713 | 1.09140199 | 1.46083098 |
| **NAALADL2-AS3** | 2.1249572 | 2.85005512 | 0.00437117 | 1.26541692 | 3.56834419 |
| **NHS-AS1** | 1.33357799 | 2.83560825 | 0.00457385 | 1.09296451 | 1.62716194 |
| **STEAP3-AS1** | 1.38068321 | 3.09133327 | 0.0019926 | 1.12530892 | 1.69401139 |
| **TOB1-AS1** | 0.68902556 | -2.8346999 | 0.00458687 | 0.53258416 | 0.89142009 |
| **APCDD1L-DT** | 1.14586406 | 2.64033562 | 0.0082824 | 1.03570874 | 1.26773523 |
| **DARS-AS1** | 1.42411087 | 2.94041026 | 0.00327778 | 1.12511633 | 1.80256183 |
| **ENO1-AS1** | 0.70215584 | -3.6774987 | 0.00023553 | 0.58155197 | 0.84777086 |
| **HHATL-AS1** | 0.70178105 | -2.8674754 | 0.00413761 | 0.55090671 | 0.89397466 |
| **LINC01436** | 1.16743515 | 2.92099307 | 0.00348918 | 1.05225281 | 1.29522564 |
| **LINC01614** | 1.15729294 | 2.74638302 | 0.00602564 | 1.04271786 | 1.28445767 |
| **LINC02522** | 1.81616733 | 3.85788692 | 0.00011437 | 1.34120174 | 2.45933455 |
| **LNCAROD** | 1.21846102 | 3.16623027 | 0.00154428 | 1.07818282 | 1.37699027 |
| **MANCR** | 1.3073737 | 4.38118801 | 1.18E-05 | 1.1596509 | 1.47391426 |
| **PSMG3-AS1** | 0.58432943 | -3.9640039 | 7.37E-05 | 0.44800609 | 0.76213448 |
| **TBL1XR1-AS1** | 1.34894082 | 3.01631291 | 0.00255869 | 1.11051664 | 1.63855387 |
| **TRAF3IP2-AS1** | 0.52580454 | -3.8009727 | 0.00014413 | 0.37745741 | 0.73245459 |
| **USP12-AS2** | 0.74354581 | -2.9672354 | 0.00300491 | 0.61136691 | 0.90430208 |
| **ZBTB46-AS1** | 0.5201618 | -2.8217447 | 0.00477632 | 0.33034689 | 0.81904296 |
| **ZNF341-AS1** | 1.40144169 | 3.28570819 | 0.00101726 | 1.14588566 | 1.71399196 |
| **ZNF674-AS1** | 0.56114117 | -3.3015004 | 0.00096169 | 0.39820496 | 0.7907471 |
| **DCST1-AS1** | 1.35742703 | 2.84817825 | 0.00439703 | 1.09998838 | 1.67511601 |
| **DHCR24-DT** | 1.34440909 | 3.04576876 | 0.00232086 | 1.11127351 | 1.6264545 |
| **LINC00472** | 1.42075244 | 3.58051988 | 0.00034291 | 1.17227734 | 1.72189416 |
| **LINC01431** | 0.68276787 | -3.8189062 | 0.00013404 | 0.56132919 | 0.83047876 |
| **LINC01705** | 1.27302692 | 4.28794808 | 1.80E-05 | 1.14003378 | 1.42153466 |
| **MHENCR** | 0.73200725 | -2.7522122 | 0.00591941 | 0.58618026 | 0.91411234 |
| **XXYLT1-AS1** | 1.45380295 | 3.31451238 | 0.00091803 | 1.16522949 | 1.81384271 |
| **DEPDC1-AS1** | 1.4157656 | 3.64992488 | 0.00026232 | 1.1746565 | 1.70636457 |
