# Supplementary material for: An immune-related lncRNA signature for the prognosis of pancreatic adenocarcinoma
Source: Aging (Albany NY). 2021 Jul 20;13(14):18806–26. doi: 10.18632/aging.203323 (PMC8351726; doi:10.18632/aging.203323)
Supplement: Supplementary Table 2 [file aging-13-203323-s003.pdf]

Supplementary Table 2. Gene set variation analysis (GSVA) of high- and low-risk groups.

	logFC	AveExpr	t	P.Value	adj.P.Val	B
HALLMARK_E2F_TARGETS	0.236067	-0.04175	4.782722	3.52E-06	9.25E-05	4.0598759
HALLMARK_ANGIOGENESIS	-0.28745	-0.01273	-4.77169	3.70E-06	9.25E-05	4.0137855
HALLMARK_G2M_CHECKPOINT	0.174587	-0.03526	4.655416	6.16E-06	0.000103	3.5328953
HALLMARK_GLYCOLYSIS	0.138911	-0.01001	4.209453	3.99E-05	0.000499	1.7765678
HALLMARK_HEDGEHOG_SIGNALING	-0.17143	0.000496	-3.87153	0.00015	0.001499	0.5428361
HALLMARK_MYOGENESIS	-0.11408	0.007783	-3.42913	0.000747	0.006223	-0.93886
HALLMARK_PANCREAS_BETA_CELLS	-0.21007	0.00353	-3.26819	0.001291	0.00922	-1.438848
HALLMARK_PEROXISOME	0.089341	-0.02105	2.930025	0.003817	0.023856	-2.419284
HALLMARK_UNFOLDED_PROTEIN_RESPONSE	0.071077	-0.03442	2.627465	0.009325	0.051808	-3.213687
HALLMARK_P53_PATHWAY	0.059283	-0.02042	2.442293	0.015536	0.077678	-3.660308
HALLMARK_MITOTIC_SPINDLE	0.062132	-0.00389	2.406108	0.017109	0.07777	-3.744023
HALLMARK_PROTEIN_SECRETION	0.08695	-0.01659	2.315119	0.021706	0.090433	-3.949337
HALLMARK_MYC_TARGETS_V2	0.125248	-0.04654	2.283943	0.023513	0.090433	-4.017968
HALLMARK_IL6_JAK_STAT3_SIGNALING	-0.09337	0.033528	-2.10634	0.036525	0.120845	-4.392124
HALLMARK_COAGULATION	-0.07013	0.011301	-2.06144	0.040662	0.120845	-4.482161
HALLMARK_ANDROGEN_RESPONSE	0.058992	-0.00348	2.055688	0.04122	0.120845	-4.493567
HALLMARK_TGF_BETA_SIGNALING	0.078125	0.01526	2.021528	0.044668	0.120845	-4.560643
HALLMARK_KRAS_SIGNALING_DN	-0.03817	0.01281	-2.021	0.044723	0.120845	-4.56167
HALLMARK_UV_RESPONSE_DN	-0.08539	-0.00536	-2.00967	0.045921	0.120845	-4.583672
HALLMARK_BILE_ACID_METABOLISM	-0.05009	-0.00775	-1.8812	0.061518	0.153795	-4.824888
HALLMARK_UV_RESPONSE_UP	-0.03275	-0.01425	-1.83857	0.067584	0.160915	-4.901547
HALLMARK_MTORC1_SIGNALING	0.074759	-0.02357	1.767002	0.078879	0.179271	-5.026433
HALLMARK_TNFA_SIGNALING_VIA_NFKB	0.073695	0.017256	1.663628	0.097882	0.212788	-5.198353
HALLMARK_ADIPOGENESIS	0.034219	-0.00511	1.567539	0.1187	0.247292	-5.349138
HALLMARK_IL2_STAT5_SIGNALING	-0.04533	0.00704	-1.52288	0.129499	0.258998	-5.41625
HALLMARK_INTERFERON_ALPHA_RESPONSE	0.069496	0.006749	1.315233	0.190062	0.357394	-5.703376
HALLMARK_ALLOGRAFT_REJECTION	-0.06749	0.00409	-1.30654	0.192993	0.357394	-5.714496
HALLMARK_HEME_METABOLISM	0.025935	-0.01057	1.229707	0.220371	0.382955	-5.809656
HALLMARK_DNA_REPAIR	0.045206	-0.03335	1.222882	0.222932	0.382955	-5.817835
HALLMARK_FATTY_ACID_METABOLISM	-0.03415	-0.01242	-1.20493	0.229773	0.382955	-5.839138
HALLMARK_SPERMATOGENESIS	-0.02513	0.035012	-1.10617	0.27009	0.43563	-5.950753
HALLMARK_MYC_TARGETS_V1	0.047904	-0.03351	1.015715	0.311094	0.486084	-6.04473
HALLMARK_EPITHELIAL_MESENCHYMAL_TRANSITION	-0.05444	0.025766	-0.94531	0.345738	0.523845	-6.112393
HALLMARK_APOPTOSIS	0.020359	0.012043	0.843576	0.399997	0.587988	-6.201654
HALLMARK_INFLAMMATORY_RESPONSE	-0.03761	0.024128	-0.82296	0.411591	0.587988	-6.218515
HALLMARK_NOTCH_SIGNALING	0.029205	0.023399	0.743809	0.457937	0.636024	-6.279404
HALLMARK_KRAS_SIGNALING_UP	-0.01816	0.008306	-0.69071	0.490613	0.66299	-6.316822
HALLMARK_APICAL_JUNCTION	-0.01775	0.012441	-0.63759	0.524533	0.690174	-6.351504
HALLMARK_PI3K_AKT_MTOR_SIGNALING	-0.01367	-0.01414	-0.59883	0.550021	0.693345	-6.375063
HALLMARK_APICAL_SURFACE	-0.02035	0.026211	-0.59185	0.554676	0.693345	-6.37915
HALLMARK_OXIDATIVE_PHOSPHORYLATION	-0.02553	-0.03656	-0.50256	0.615874	0.751066	-6.427213
HALLMARK_ESTROGEN_RESPONSE_LATE	0.011495	-0.00632	0.401549	0.688481	0.813378	-6.472152
HALLMARK_CHOLESTEROL_HOMEOSTASIS	0.01349	-0.00238	0.386589	0.699505	0.813378	-6.477956
HALLMARK_REACTIVE_OXYGEN_SPECIES_PATHWAY	-0.01355	-0.00738	-0.35706	0.721456	0.819836	-6.488767
HALLMARK_XENOBIOTIC_METABOLISM	-0.00799	-0.00284	-0.25988	0.795246	0.883607	-6.518288
HALLMARK_COMPLEMENT	-0.00719	-0.00372	-0.19603	0.844803	0.901439	-6.532625
HALLMARK_WNT_BETA_CATENIN_SIGNALING	0.007175	-0.01009	0.192768	0.847352	0.901439	-6.533249
HALLMARK_INTERFERON_GAMMA_RESPONSE	-0.00104	0.017869	-0.02033	0.983801	0.995271	-6.551351
HALLMARK_HYPOXIA	0.000247	-0.00195	0.008861	0.992939	0.995271	-6.551516
HALLMARK_ESTROGEN_RESPONSE_EARLY	-0.00015	0.003169	-0.00593	0.995271	0.995271	-6.551537
